# Comparing mTOR inhibitor Rapamycin with Torin-2 within the RIST molecular-targeted regimen in neuroblastoma cells

**DOI:** 10.7150/ijms.48393

**Published:** 2021-01-01

**Authors:** Rebecca Waetzig, Marie Matthes, Johannes Leister, Gina Penkivech, Tilman Heise, Selim Corbacioglu, Gunhild Sommer

**Affiliations:** Department of Pediatric Hematology, Oncology and Stem Cell Transplantation, University Hospital of Regensburg, Franz-Josef-Strauss Allee 11, 93053, Regensburg, Germany.

**Keywords:** mTOR inhibitor, Torin-2, Rapamycin, neuroblastoma, ATP competitive mTOR inhibitors, combination therapy

## Abstract

The prognosis for patients with relapsed or refractory high-risk neuroblastoma remains dismal and novel therapeutic options are urgently needed. The RIST treatment protocol has a multimodal metronomic therapy design combining molecular-targeted drugs (Rapamycin and Dasatinib) with chemotherapy backbone (Irinotecan and Temozolomide), which is currently verified in a phase II clinical trial (NCT01467986). With the availability of novel and more potent ATP competitive mTOR inhibitors, we expect to improve the RIST combination therapy. By comparing the IC_50_ values of Torin-1, Torin-2, AZD3147 and PP242 we established that only Torin-2 inhibited cell viability of all three MycN-amplified neuroblastoma cell lines tested at nanomolar concentration. Single treatment of both mTOR inhibitors induced a significant G_1_ cell cycle arrest and combination treatment with Dasatinib reduced the expression of cell cycle regulator cyclin D1 or increased the expression of cell cycle inhibitor p21. The combinatorial index depicted for both mTOR inhibitors a synergistic effect with Dasatinib. Interestingly, compared to Rapamycin, the combination treatment with Torin-2 resulted in a broader mTOR pathway inhibition as indicated by reduced phosphorylation of AKT (Thr308, Ser473), 4E-BP (Ser65), and S6K (Thr389). Furthermore, substituting Rapamycin in the modified multimodal RIST protocol with Torin-2 reduced cell viability and induced apoptosis despite a significant lower Torin-2 drug concentration applied. The efficacy of nanomolar concentrations may significantly reduce unwanted immunosuppression associated with Rapamycin. However, at this point we cannot rule out that Torin-2 has increased toxicity due to its potency in more complex systems. Nonetheless, our results suggest that including Torin-2 as a substitute for Rapamycin in the RIST protocol may represent a valid option to be evaluated in prospective clinical trials for relapsed or treatment-refractory high-risk neuroblastoma.

## Introduction

Neuroblastoma, derived from primitive nervous sympathetic cells, is the most common solid tumor of childhood and constitutes 7 % of all pediatric cancers 1,2. Neuroblastoma is an ambiguous disease with spontaneous remissions in infants with stage 4s while children over 1 year of age with metastatic disease have a persistently poor outcome.^3^ Amplification of the MycN gene is found predominantly in advanced stage diseases and is associated with rapid tumor progression and poor prognosis 2,4-6. Despite intensive therapy the prognosis of patients with high-risk relapsed or refractory neuroblastoma (rNB) comprising up about 60% of cases remains dismal 2,7. Therefore, development of new treatment protocols is urgently needed.

The RIST protocol for rNB is currently being evaluated in a phase II prospective randomized clinical trial (ClinicalTrials.gov Identifier: NCT01467986) 8. This novel metronomic multimodal treatment protocol combines two molecular-targeted drugs, the mTOR-inhibitor Rapamycin and the multi-kinase inhibitor and immunosuppressant Dasatinib with conventional chemotherapy, consisting of the topoisomerase inhibitor irinotecan and the alkylating agent temozolomide. The “pre-treatment” with Rapamycin and Dasatinib is anticipated to have an apoptotic, chemo-sensitizing and cell cycle synchronizing effect 9. The metronomic therapy is expected to reduce toxicity 10, prevent drug resistance, and to alter the tumor microenvironment in a more anti-tumorigenic manner 9. With targeting key cancer promoting pathways by synergistically acting drugs, the combination therapy is expected to enhance efficacy compared to the monotherapy. Combination therapy is assumed to reduce drug resistance and dosage, induce anti-tumor effects such as suppressing growth and metastatic potential, arrest mitotically active cells, reduce the cancer stem cell population and induce apoptosis 11. In a compassionate use setting, the RIST therapy revealed promising results in rNB patients with an overall survival of 55 % and a tolerable adverse event profile 8.

Dysregulation of the PI3K/AKT signaling pathway is common in human malignancies 12-14, promoting neoplastic transformation 13, altering cell growth and survival 12. Different studies demonstrated that the PI3K/AKT/mTOR pathway plays an important role in neuroblastoma pathogenesis and inhibition of this signaling pathway by mTOR inhibitors were effective in neuroblastoma models 7. It has been shown that inhibiting PI3K/AKT/mTOR signaling cascade induces downregulation of MycN expression and reduces the growth of neuroblastoma cells *in vitro* and *in vivo*. In high-risk neuroblastoma, activation of the PI3K pathway is common 15 and phosphorylation-mediated activation of AKT was shown to be frequently triggered in neuroblastoma 16. Furthermore activation of AKT by phosphorylation is associated with poor prognosis in primary neuroblastoma and correlates with amplification of the oncogene MycN, a well-known marker of an aggressive phenotype 16. Hence, agents targeting this pathway are potential treatment options of high-risk and relapsed neuroblastoma 17-19.

Besides the activation of mammalian target of Rapamycin (mTOR) by the PI3K/AKT pathway, the protein kinase mTOR integrates various other cellular signals to control cell proliferation, growth and metabolism depending on growth factor signaling, as well as nutrient, energy and oxygen supply 14. In more than 70% of cancer, mTOR is activated and promotes neoplastic growth and progression 20. Therefore, mTOR is a promising target for cancer therapy. mTOR serves as core component for two distinct protein complexes, mTOR complex 1 (mTORC1) and mTOR complex 2 (mTORC2) 21, which regulate different cellular processes 22. The mTORC1 is one of the major downstream effectors of the PI3K/AKT signaling cascade 23. Currently, the allosteric mTOR inhibitor Rapamycin is part of the RIST therapy protocol 8. It has been shown that Rapamycin inhibits the mTORC1 incompletely 23-25 and that inhibition of mTORC2 is cell-line dependent and occurs after long-term treatment 26,27. Additionally, Rapamycin-mediated mTORC1 inhibition causes activation of PI3K/AKT by suppression of the negative feedback loop 28,29, promotes survival through AKT, and partly contributes to the known ineffectiveness of Rapamycin treatment 28. The limited success of the allosteric inhibitor Rapamycin as an anti-cancer drug led to the development of ATP competitive mTOR inhibitors 30,31. This new drug class potently inhibits both mTOR complexes and consequently may prevent AKT feedback activation 32.

In this study, we hypothesized that inhibition of the mTOR pathway by ATP competitive mTOR inhibitors enhances the efficacy of the conventional RIST therapy. Therefore, we performed a comparative study of Rapamycin with four different ATP competitive mTOR inhibitors, namely Torin-1, Torin-2, AZD3147 and PP242. Our results demonstrate that in the combination drug treatment Torin-2 and protein kinase inhibitor Dasatinib act synergistically. Furthermore, signaling pathway analysis revealed that inhibition of the mTOR pathway by Rapamycin was incomplete and leaky compared to Torin-2. Taken together, the cell-based studies demonstrate that at significant lower Torin-2 drug doses, the alternative 'T'-IST protocol reduced neuroblastoma cell viability as effective as the conventional RIST protocol.

## Materials and Methods

### Cell culture and drug treatment

All neuroblastoma cell lines were purchased from Deutsche Sammlung für Mikroorganismen und Zellkulturen (DSMZ). The cells were cultivated for not more than 40 passages at 37 °C in a humidified 5 % CO_2_ atmosphere. The cell line Kelly was cultured in RPMI 1640 (Gibco®, Cat. No. 11875093) supplemented with 15 % fetal bovine serum (FBS, Biochrom), 1% penicillin/streptomycin (Biochrom, Cat. No. A2212) and 2 mM L-glutamine (Gibco®, Cat. No. 25030081). The cell line IMR-32 was cultured in RPMI 1640 with supplemented with 20% FBS, 1% penicillin/streptomycin and 1% non-essential amino acids (MEM NEAA, Gibco®, Cat. No. 11140050). The cell line SK-N-BE(2) was cultured in EMEM (Lonza, Cat. No. 12-125F)/Ham´s F12 (Biochrom, Cat. No. F0815) 1:1 supplemented with 10% FBS, 1% penicillin/streptomycin and 2 mM L-glutamine. All cell lines were tested periodically for mycoplasma contamination.

AZD3147 (Cat. No. 5615), PP242 (Cat. No. 4257), Torin-1 (Cat. No. 4247), Torin-2 (Cat. No. 4248) and SN-38 (active metabolite of Irinotecan, Cat. No. 2684) were purchased from Tocris Bioscience. Rapamycin (Cat. No. R-5000) and Dasatinib (Cat. No. D-3307) were purchased from LC Laboratories. Temozolomide (TMZ) was purchased from BioVision (Cat. No. 2226-10). All drugs were dissolved in DMSO (Sigma, Cat. No. D2650) following the manufacturer´s instructions and stored at -20 °C. Cells were treated with indicated concentrations and for indicated time-periods. As control, cells were treated with appropriate DMSO concentrations. The applied drug concentrations for single or combination treatment are listed in Table 3 and Table 4, respectively. The treatment protocol has been performed as shown in Figure 12.

### Determination of the IC_50_ by applying the MTT cell viability assay

The MTT assay was performed as described in David Morgan's “Polyamine Protocols. Methods in Molecular Biology”^33^ with minor changes of the protocol as described: 24 h after plating, 50% confluent cells were treated in concentration series including the expected IC_50_ concentration of the respective substance. After 72 h of incubation the MTT reaction and photometric analysis was implemented. The cell culture medium was aspirated with a vacuum pipette before applying 100 µL MTT working solution (MTT stock solution: 20 mg/ml MTT (Sigma, Cat. No. M5655) in 1× PBS (D8537, Sigma); MTT working solution: 1:5-dilution of the stock solution with RPMI 1640 medium without phenol red). The reaction was stopped after 2 h at 37 °C with 100 µL isopropanol (70 %, Cat. No. 3889017). The photometric analysis at λ = 560 nm was performed in a TECAN Plate Reader after an additional 30 min incubation at room temperature (RT). The viabilities were normalized to DMSO controls. For the identification of the IC_50_ values all experiments were performed in three replicates and repeated in at least three independent experiments.

### Calculation of the Combination Index (CI)

To describe the effect of the combination treatments with Dasatinib/Rapamycin or Dasatinib/Torin-2 we calculate the Combination Index (CI) applying the Chou-Talalay-Method 34 as followed:


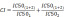


The calculations were done with the software CalcuSyn. We used normalized viability values determined by MTT assays as described above. For the calculation the viability values were converted to the “Fraction Affected” (FA) 35 as followed:


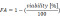


For the calculation, a constant dilution factor for both drugs is necessary. To test different concentration ranges we used various dilution factors (such as 1.5, 2.0 or 2.5). Three replicates were performed for each experiment. For calculation of the CI we used the results of at least two independent experiments.

The calculation of the Dose Reduction Factor (DRF) was done as followed:


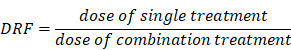


### Immunoblot analysis

24 h after plating, 50% confluent cells were treated. The drug concentrations applied are listed in Table 3 (single treatment) respectively Table 4 (combination treatment). After 6 h, 24 h or 72 h for single or combination treatment, respectively at day 8 of the multimodal RIST and TIST treatment, the cells were harvested. The whole cell lysates were separated by gel electrophoresis and the proteins were transferred to nitrocellulose membranes (Amersham^TM^ Protran^TM^ 0,45 µm, GE Healthcare Life Science, Cat. No. 10600002). After the transfer, the membranes were incubated with primary antibodies (1:1000, respectively 1:2000 for LC3B and GAPDH antibody). The primary antibodies AKT (pan, #4691), phospho-AKT (Ser473, #4060), phospho-AKT (Thr308, #13038), 4E-BP1 (#9644), phospho-4E-BP1 (Ser65, #9456), MycN (#9405), p70S6 kinase (#9202), phospho-p70S6 kinase (Thr389, #9234), Src kinase (#2108), phospho-Src family (Tyr416, #2101), caspase-3 (#9665) and p21 Waf1/Cip (DCS60, #2946) were purchased from Cell Signaling. The LC3B antibody (#NB100-2220SS) was purchased from NovusBio, the PARP-1 antibody (#1072-1) was purchased from Epitomics and the GAPDH antibody (#Sc-47724) was purchased from Santa Cruz. ImageJ was used for quantification of the protein expression.

### Cell cycle analysis

For the cell cycle analysis, we followed the univariate cell cycle analysis protocol for the MACSQuant® Analyzer (Miltenyi) with minor changes: We used 20 µg propidium iodide (Sigma, #P4864) and 0,2 mg RNase A (Thermo Fisher, #EN0531). The measurement of the fluorescence signal was done with the MACSQuant Analyzer10 (Miltenyi).

### Statistical analysis

For the IC_50_ value of a single drug we created a graph of cell viability versus the logarithm of the molar drug concentration in GraphPad Prism 6 using the viability values of the MTT assay: First the cell viabilities were transformed to logarithmic values and then normalized to get a range between 0 and 100% viability. Afterwards, a non-linear regression analysis was performed. The results are presented as the mean ± standard deviation (SD). To evaluate the effect on viability reduction by combination treatment or multimodal treatments compared to the control an unpaired t-test was performed. For the *P* value consistent SD were not assumed. For statistical significance, the Holm-Sidak method and a significance level of α = 5,00% were used.

## Results

### Inhibition of cell viability by Torin-2 compares to Rapamycin

Firstly, the half maximal inhibitory concentration (IC_50_) of four different ATP competitive mTOR inhibitors was determined by performing MTT assays for the neuroblastoma (NB) cell lines Kelly and IMR-32 (Table 1, Fig. S1 and S2). The IC_50_ values for Torin-1, AZD3147 and PP242 varied widely in the two cell lines tested. In contrast, the IC_50_ value of Torin-2 was in both cell lines in the low nanomolar range (Kelly: 11.69 nM, IMR-32: 29.67 nM, Table 1, Fig. S2A and B). Due to the consistent effect of Torin-2 in two cell lines and the large variability of sensitivity to the inhibitors Torin-1, AZD3147, and PP242, further studies were performed with Torin-2. Moreover, we confirmed in an additional NB cell line that Torin-2 is effective in nanomolar range by establishing an IC_50_ of 28.52 nM for SK-N-BE cells (2) (Fig. S2C). The comparison of the IC_50_ values demonstrated a thousand-fold lower IC_50_ for Torin-2 compared to Rapamycin in three different NB cell lines (Table 1). Hence the cell viability of all NB cell lines tested is impaired by treatment with low nanomolar concentrations of the ATP-competitive mTOR inhibitor Torin-2.

### Induction of a G_1_ cell cycle arrest by Torin-2

**Figure 1:**
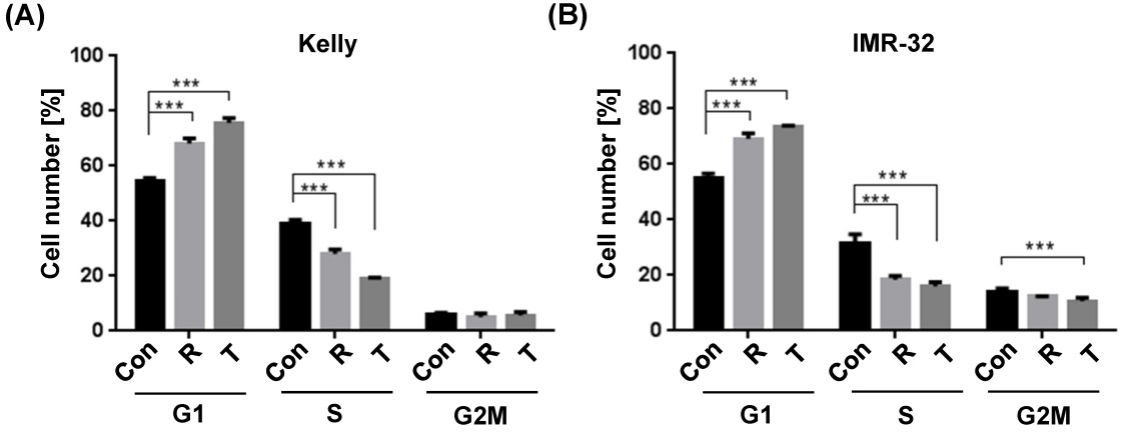
Cell cycle analysis comparing Rapamycin (R) and Torin-2 (T) at the corresponding IC_50_ concentrations in neuroblastoma cell line (A) Kelly [R: 30 μM, T: 12 nM] and (B) IMR-32 [R: 40 μM, T: 30 nM]. Con: DMSO-treated control cells. G1-, S-, and G2M cell cycle phases. Cells were seeded 24h before treatment with Rapamycin and Torin 2, respectively, and DMSO concentrations applied as vehicle for control cells. After an incubation of 72h, the cells were harvested, prepared and stained following the univariate cell cycle analysis protocol for the MACSQuant® Analyzer (Miltenyi) with minor changes described in Materials and Methods.

It has been shown earlier that the allosteric mTOR inhibitor Rapamycin inhibits the progression of the cell cycle from G_1_ to S phase 36. In an effort to study the underlying cellular mechanisms whereby Torin-2 impairs NB cells viability, we tested the impact of Torin-2 on cell cycle progression. Our data confirm the cell cycle inhibiting effect of Rapamycin and demonstrate for the first time that Torin-2 induces a G_1_ arrest in NB cells as shown by a significant increase in cell counts in G_1_ and a significantly decrease in the S phase (Fig. 1A and B).

### Effect of single drug treatment with Torin-2 or Rapamycin on the expression of cell cycle regulator cyclin D1 and p21 as well as autophagy marker LC3B-II

**Figure 2:**
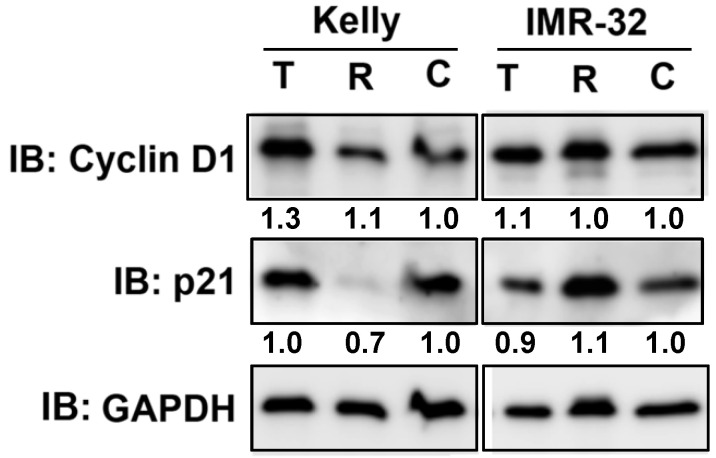
Immunoblot for cell cycle regulator Cyclin D1 and p21. Kelly cells were treated with IC_50_ concentration of Torin-2 (T) [IC_50_ = 12 nM] or Rapamycin (R) [IC_50_ = 30 μM]. IMR-32 cells were treated with IC_50_ concentrations of Torin-2 [IC_50_ = 30 nM] or Rapamycin [IC_50_ = 40 μM]. C: DMSO-treated control cells. GAPDH was applied as loading control. Numbers below bands state protein expression normalized to GAPDH. Cells were harvested 24 h (Cyclin D1), respectively 72 h (p21) after treatment.

We have shown that Rapamycin and Torin-2 treatment at IC_50_ concentrations (Table 1) induced a G_1_ arrest in both NB cell lines (Fig. 1). Next, we tested whether those drug concentrations change the expression of two important cell cycle regulators cyclin D1 and cyclin-dependent kinase inhibitor p21 regulating the cell cycle progression from G_1_ to S phase. As shown, cyclin D1 expression was increased by Torin-2 treatment in Kelly but barely in IMR-32 cells and p21 expression was reduced by Rapamycin in Kelly, but only slightly increased in IMR-32 cells (Fig. 2). Those data suggest that the observed G_1_ arrest in single drug treated Kelly and IMR-32 cells did not result from reduced cyclin D1 or increased p21 expression.

Because ATP competitive mTOR inhibitors such as Rapamycin can induce autophagy 37, we asked next whether single drug treatment induces the expression of autophagy marker LC3B-II 38. Torin-2 treatment at IC_50_ concentrations or even several magnitudes higher did not increase the expression of LC3B-II in Kelly nor IMR-32 cells, whereas Rapamycin treatment caused an increase of LC3B-II in both NB cell lines at concentrations slightly higher than the IC_50_ value (Fig. 4). In summary, in both cell lines the expression of autophagy marker LC3B-II was not induced at IC_50_ concentrations. However, increasing Rapamycin but not Torin-2 concentrations lead to enhanced LC3B-II expression suggesting that autophagy induced by Rapamycin but not Torin-2 treatment contributes to the observed G_1_ arrest 39 in both cell lines.

### Reduced induction of apoptosis by Torin-2 compared to Rapamycin

**Figure 3:**
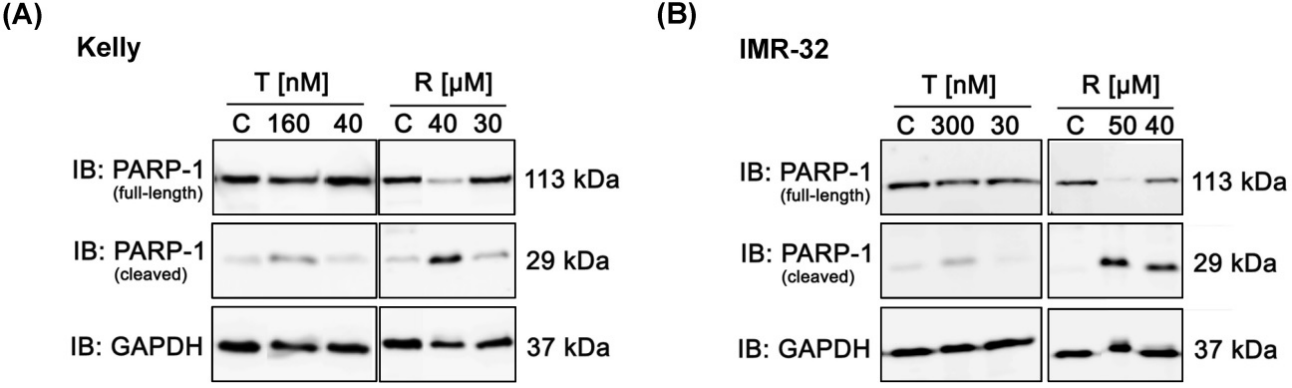
Immunoblot (IB) analysis of apoptosic marker expression (PARP cleavage) comparing Rapamycin (R) and Torin-2 (T) in neuroblastoma cell line (A) Kelly and (B) IMR-32. C: Vehicle (DMSO)-treated control cells. GAPDH served as loading control. To test for the induction of apoptosis, neuroblastoma cells were seeded 24h before treatment with Torin-2 or Rapamycin at the corresponding IC_50_ and higher concentrations in (A) Kelly [T: IC_50_ = 12nM, R: IC_50_ = 30μM] and (B) IMR-32 [T: IC_50_ = 30nM, R: IC_50_ = 40 μM]. 72 h after treatment cells were harvested.

**Figure 4:**
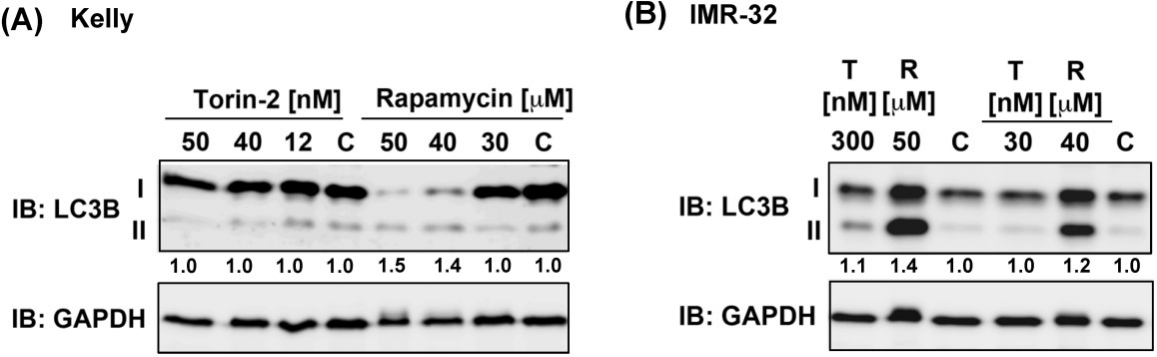
Immunoblot for autophagy marker LC3B-II. (A) Kelly cells were treated with IC_50_ and increasing concentrations of Torin-2 [IC_50_ = 12nM] or Rapamycin [IC_50_ = 30μM]. (B) IMR-32 cells were treated with IC_50_ and increasing concentrations of Torin-2 (T) [IC_50_ = 30nM] or Rapamycin (R) [IC_50_ = 40μM]. C: DMSO-treated control cells. Numbers below bands state ratio between autophagy marker LC3B-II/I. GAPDH was applied as loading control.

Further, we analyzed whether treatment with Rapamycin or Torin-2 induces apoptosis in NB cells. Therefore, we monitored the apoptosis-dependent processing of PARP-1 upon treatment with increasing concentrations of Rapamycin or Torin-2 in Kelly and IMR-32 cells. Treatment of cells with Rapamycin concentrations slightly above the IC_50_ value (Rapamycin in Kelly (IC_50_: 27 µM) or IMR-32 (IC_50_: 37 µM)) clearly induced PARP-1 cleavage (Fig. 3A and B). In contrast, treatment of NB cells with even a high dose - more than tenfold - of Torin-2 (160 nM) only marginally induced PARP-1 cleavage (Fig. 3A and B). We therefore conclude that nanomolar Torin-2 concentrations do not induce apoptosis and impair cell viability mainly through induction of a G_1_ cell cycle arrest.

### Synergistic effect of combination treatment with Dasatinib and Torin-2

Next, we tested whether the drug combination Dasatinib and Torin-2 (D+T) inhibits cell viability as effective as the combination of Dasatinib and Rapamycin (D+R). Accordingly, we first determined the IC_50_ values for Dasatinib in Kelly (IC_50_: 9.47 µM), IMR-32 (IC_50_: 1.53 µM), and SK-N-BE(2) (IC_50_: 25.73 µM) (Fig. S3, Table S1).

Subsequently, we determined the combinatorial index (CI) for the combination treatments (Dasatinib/Rapamycin (D+R) and Dasatinib/Torin-2 (D+T)) by using the Chou-Talalay method 34. Both combination treatments showed a synergistic effect in Kelly (D+R: CI = 0.23; D+T: CI = 0.60, Table 2) and in IMR-32 (D+R: CI = 0.20; D+T: CI = 0.17, Table 2). In SK-N-BE(2) the combination Dasatinib/Rapamycin was synergistic (D+R: CI = 0.75, Table 2) as well, whereas Dasatinib/Torin-2 showed a slight antagonistic effect (D+T: CI = 1.25, Table 2). Due to the increased efficacy of all drug combination treatments compared to single treatments, it was possible to calculate a dose reduction factor (DRF) for each drug (Table S2 and S3). Based on the combinatorial effects and the calculated DRF, all drugs were used at concentrations below their IC_50_ applied for the single drug treatment (compare Table 3 and Table 4) in the following combination treatment experiments.

**Figure 5:**
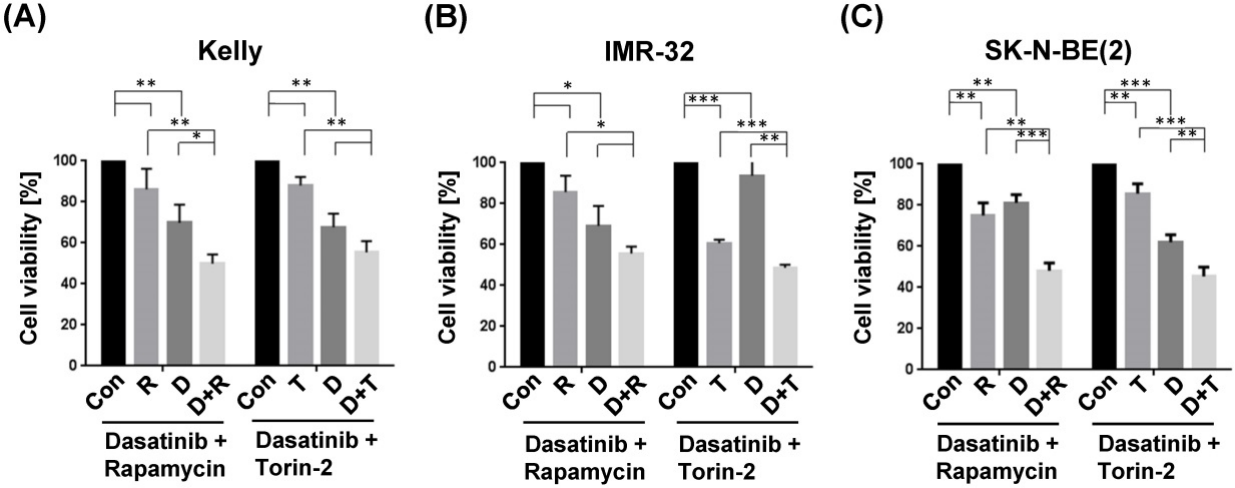
Viability tests of single and combination drug treatment applying tyrosine kinase inhibitor Dasatinib (D), and mTOR inhibitors Rapamycin (R) or Torin-2 (T) in neuroblastoma cell line (A) Kelly, (B) IMR-32, and (C) SK-N-BE(2). Con: Vehicle (DMSO)-treated control cells. Neuroblastoma cells were seeded 24h before treatment. For single treatments the concentrations of the combination treatments was used: (A) Kelly [D+R = 5μM + 2μM; D+T = 1.5μM + 9nM]; (B) IMR-32 [D+R = 0.05μM + 0.13μM; D+T = 1μM + 6nM]; (C) SK-N-BE(2) [D+R = 20μM + 5μM; D+T = 12.5μM + 15nM]. After an incubation of 72h, the cell viability was tested by applying the MTT test as described under Methods. The cell viability was normalized to control-treated cells.

The combination treatment with Dasatinib/Rapamycin (D+R) induced a significantly stronger inhibition of cell viability compared to the treatment with both single drugs in Kelly (Fig. 5A; (D): 5 µM, (R): 2 µM) and SK-N-BE(2) (Fig. 5C; (D): 20 µM, (R): 5 µM), however, in IMR-32 cells the viability was not significant but strongly reduced (p=0.20, Fig. 5B; (D): 0.05 µM, (R): 0.13 µM). The combination treatment with Dasatinib/Torin-2 (D+T) induced a significantly stronger inhibition of cell viability compared to the treatment with both single drugs in IMR-32 (Fig. 5B; (D): 1 µM, (T): 6 nM) and SK-N-BE(2) (Fig. 5C; (D): 12.5 µM, (T): 15 nM), however, in Kelly cells the viability was not significant but strongly reduced (p=0.07, Fig. 5A; (D): 1.5 µM, (T): 9 nM) in the combination treatment compared to the single treatment with Torin-2.

Taken together, both Dasatinib/Rapamycin and Dasatinib/Torin-2 treatments inhibited the cell viability of NB cells at reduced concentrations compared to single drug treatments.

### Combination treatments impact the expression of cell cycle regulator cyclin D1 and p21

**Figure 6:**
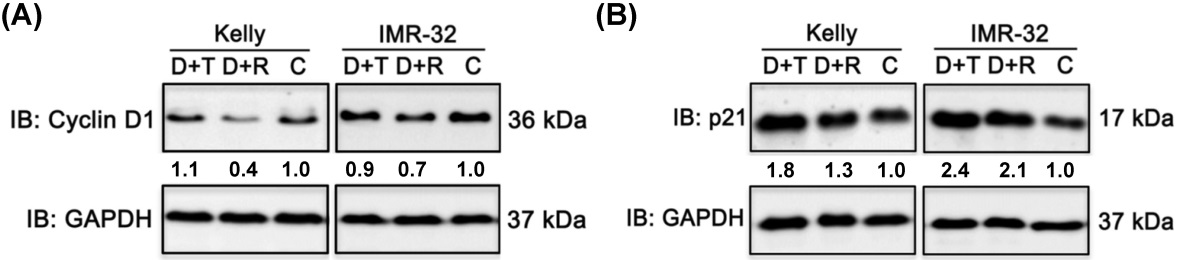
Immunoblot (IB) analysis of cell cycle regulator (A) cyclin D1 expression and (B) cyclin-dependent kinase inhibitor p21 expression comparing Dasatinib combined with Torin-2 (D+T) or Rapamycin (D+R) treatment in neuroblastoma cell line Kelly and IMR-32. C: Vehicle (DMSO)-treated control cells. GAPDH served as loading control. Protein expression corrected to GAPDH is indicated in numbers below bands. Cells were seeded 24 h before drug treatment with a combination of Dasatinib with Torin-2 or Rapamycin, respectively. Drug concentration used for the combination treatment in Kelly [D+T = 1.5 μM + 9 nM; D+R = 5 μM + 2 μM;] and IMR-32 [D+T = 1 μM + 6 nM; D+R = 0.05 μM + 0.13 μM]. Cells were harvested 24 h (cyclin D1), respectively 72 h (p21) after treatment.

We have shown that Rapamycin as well as Torin-2 treatment induced a G_1_ arrest in NB cell line Kelly and IMR-32 (Fig. 1). In a next step we wanted to test whether combination treatments with Dasatinib inhibit the cell cycle progression as well. Therefore, we tested the expression of cell cycle regulators cyclin D1 after 24 h and p21 after 72 h in NB cells treated with Dasatinib/Rapamycin (D+R) and Dasatinib/Torin-2 (D+T) using immunoblot analysis. As shown, D+R treatment reduced cyclin D1 expression in both cell lines compared to the control-treated cells (Fig. 6A). On the contrary, we did not detect any reduction of cyclin D1 expression upon D+T treatment in Kelly and only a slight reduction in IMR-32 cells (Fig. 6A). In both NB cell lines, the D+T treatment resulted in a significant increase in p21 expression compared to untreated controls (Fig. 6B). The effect of D+R treatment on p21 expression was approximately strong in IMR-32 cells and less pronounced in Kelly cells compared to D+T treatment. We concluded that both mechanisms - inhibition of cyclin D1 and induction of p21 expression - might contribute to induction of the G_1_ cell cycle arrest in NB cells by Dasatinib/Rapamycin and Dasatinib/Torin-2 combination treatment.

### Both combination treatments only marginally induced apoptosis or autophagy

**Figure 7:**
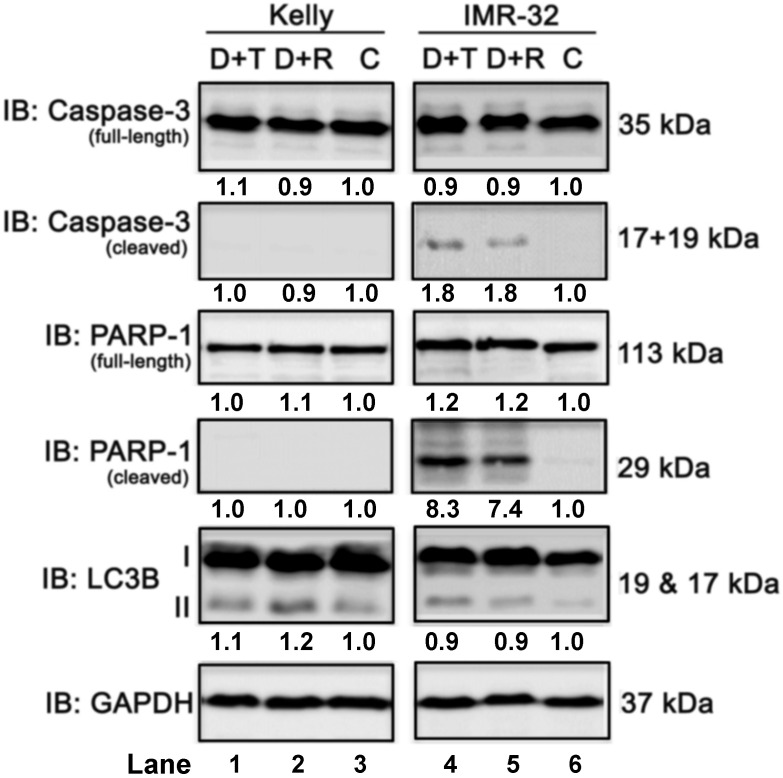
Immunoblot (IB) analysis of apoptosis (caspase-3 and PARP) and autophagy (LC3B) marker expression comparing Dasatinib combined with Torin-2 (D+T) or Rapamycin (D+R) treatment for 72h in neuroblastoma cell line Kelly and IMR-32. C: Vehicle (DMSO)-treated control cells. GAPDH served as loading control. Numbers below bands state protein expression normalized to GAPDH. Numbers below bands of autophagy marker LC3B represent the ratio between LC3B-II/I. Cells were seeded 24h before drug treatment with a combination of Dasatinib with Torin-2 or Rapamycin, respectively. Drug concentrations used for the combination treatments in Kelly [D+T = 1.5 μM + 9 nM; D+R = 5 μM + 2 μM;] and IMR-32 [D+T = 1 μM + 6 nM; D+R = 0.05 μM + 0.13 μM]. Cells were harvested 72h after treatment.

Next, we investigated the effect of the combination treatment with Dasatinib/Rapamycin (D+R) or Dasatinib/Torin-2 (D+T) on induction of apoptosis. In Kelly cells both treatment combinations did not induce the cleavage of caspase-3 or PARP-1 (Fig. 7, compare lane 1-3), which was applied as marker for apoptosis, however, both D+R and D+T induced apoptosis in IMR-32 cells (Fig. 7, lane 4-6). Because ATP competitive mTOR inhibitors, such as Rapamycin, can induce autophagy 37, we asked next whether D+R or D+T treatment induces the expression of the autophagy marker LC3B-II 38. Both combination treatments caused a minor increase of LC3B-II in Kelly cells only (Fig. 7, lane 4-6). Taken together, D+R and D+T combination treatments marginally induced apoptosis in IMR-32, but autophagy in Kelly cells.

### Combination treatment with Dasatinib/Torin-2 results in stronger mTOR pathway inhibition compared to Dasatinib/Rapamycin

To determine and compare how Dasatinib/Torin-2 (D+T) or Dasatinib/Rapamycin (D+R) combination treatment affects the mTOR signaling pathway, we analyzed the phosphorylation status of downstream targets of mTORC1 (4E-BP, Ser65 and ribosomal protein kinase S6, Thr389) and mTORC2 (AKT, Ser473), as well as AKT phosphorylation at Thr308 by PDK-1, a kinase activated by PI3K signaling 31 in NB cell lines Kelly and IMR-32 6 and 24 h after treatment using immunoblot analysis.

**Figure 8:**
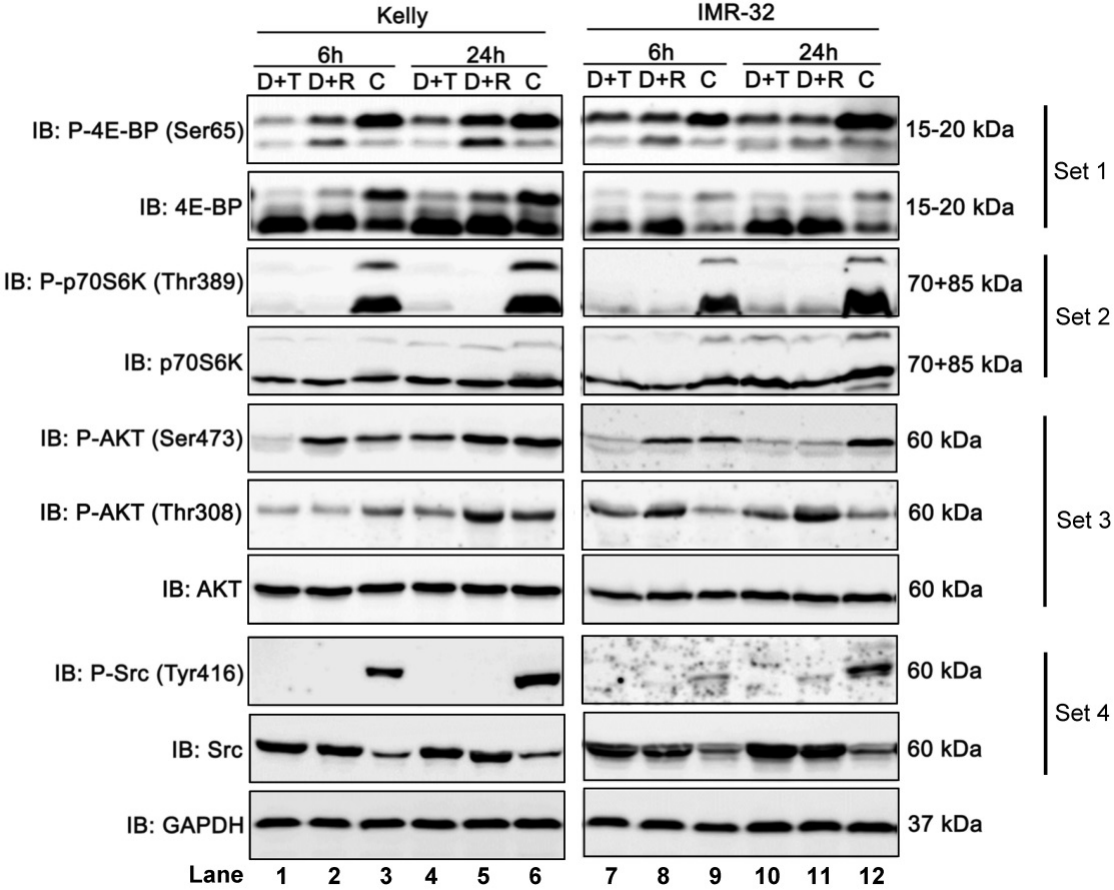
Immunoblot (IB) analysis comparing Dasatinib combined with Rapamycin (D+R) or Torin-2 (D+T) in neuroblastoma cell line Kelly and IMR-32. C: Vehicle (DMSO)-treated control cells. GAPDH served as loading control. Cells were seeded 24 h before drug treatment with a combination of Dasatinib with Torin-2 or Rapamycin, respectively. Drug concentrations used for the combination treatments in Kelly [D+T = 1.5 μM + 9 nM; D+R = 5 μM + 2 μM;] and IMR-32 [D+T = 1μM + 6nM; D+R = 0.05 μM + 0.13 μM]. The cells were harvested 6 h or 24 h after treatment.

It is shown that phosphorylation of 4E-BP at Ser65 (P-4E-BP, Ser65), a well-known regulator of protein synthesis and downstream target of mTORC1, was more strongly inhibited by D+T compared to D+R treatment in Kelly (Fig. 8, set 1, lane 1-6), but only modestly in IMR-32 (Fig. 8, set 1, lane 7-12). In both cell lines, D+T and D+R treatment dramatically reduced phosphorylation of ribosomal protein kinase S6 at Thr389 (P-p70S6K, Thr389), which is another well-known mTORC1 target (Fig. 8, set 2, lane 1-12).

Further, we investigated the phosphorylation of AKT at Ser473, representing a downstream target of mTORC2. The combination treatment with D+R did not reduce phosphorylation at Ser473 after 6 h in both cell lines (Fig. 8, set 3, lane 2 and 8). The effect at 24 h was cell line-specific: In Kelly cells the phosphorylation was not reduced by D+R compared to the control (Fig. 8, set 3, lane 5 and 6), while there was a clear reduction in IMR-32 cells (Fig. 8, set 3, lane 11 and 12). It has been shown that Rapamycin can affect mTORC2 in a cell line-specific manner and after prolonged treatment.^26^ In contrast, AKT (Ser473) was effectively inhibited by D+T treatment at 6 and 24 h in both cell lines (Fig. 8, set 3, compare lane 1 and 3, lane 4 and 6, lane 7 and 9, lane 10 and 12).

Besides the inhibition of AKT phosphorylation at Ser473 we also assessed the effect of D+T or D+R treatment on phosphorylation site Thr308, a direct target site of protein kinase PDK-1. The D+T treatment impaired Thr308 phosphorylation at 6 and 24 h in Kelly cells (Fig. 8, set 3, compare lane 1 and 3, lane 4 and 6), whereas D+R treatment only inhibited Thr308 phosphorylation at 6 h after treatment and slightly increased after 24 h of treatment compared to control cells (Fig. 8, set 3, compare lane 2 and 3, lane 5 and 6). Puzzlingly, in IMR-32 cells D+T as well as D+R treatment induced Thr308 phosphorylation (Fig. 8, set 3, compare lane 7, 8 with 9, and lane 10, 11 with 12).

As expected, in both cell lines, D+T and D+R treatments inhibited the phosphorylation of Src kinase at Tyr416 (Fig. 8, set 4, lane 1-12) by the tyrosine-kinase inhibitor Dasatinib.

In summary, Dasatinib/Torin-2 treatment potently inhibits both mTOR complexes, whereas the Dasatinib/Rapamycin treatment inhibited mainly mTORC1 and - in a cell line dependent manner - also mTORC2 after prolonged drug treatment.

### Both combination treatments strongly impair MycN expression in IMR-32 cells

**Figure 9:**
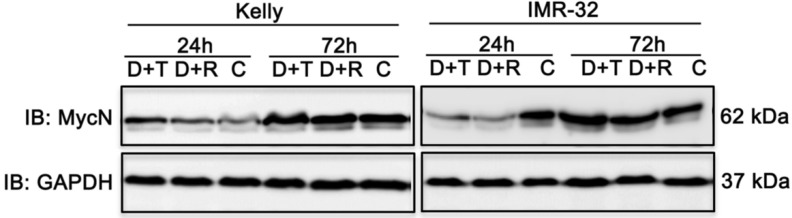
Immunoblot (IB) analysis of oncogene expression MycN comparing Dasatinib combined with Rapamycin (D+R) or Torin-2 (D+T) treatment for 24 h and 72 h in neuroblastoma cell line Kelly and IMR-32. C: Vehicle (DMSO)-treated control cells. GAPDH served as loading control. Cells were seeded 24 h before drug treatment with a combination of Dasatinib and Torin-2 or Rapamycin, respectively. Drug concentrations used for the combination treatment in Kelly [D+T = 1.5 μM + 9 nM; D+R = 5 μM + 2 μM;] and IMR-32 [D+T = 1 μM + 6 nM; D+R = 0.05 μM + 0.13 μM]. The cells were harvested 24 h and 72 h after treatment.

MYC transcription factors significantly stimulate cell growth and differentiation explaining why increased expression of MYCN leads to progression of cell cycle and inhibition of apoptosis of neuroblastoma cell lines 2. Hence, therapeutic reduction of MycN expression is a noteworthy approach to improve treatment of neuroblastoma 40 as supported by studies in mouse models of MYCN-driven neuroblastoma. In those studies, inhibition of PI3K/AKT/mTOR induced destabilization of the MYCN oncoprotein and triggered anti-tumor effects 41,42. Therefore, we tested the impact of D+R and D+T treatment on MycN expression in Kelly and IMR-32 cells 24 h and 72 h after treatment using immunoblot analysis. Whereas both treatments had no effect on MycN expression in Kelly cells, a marked decrease of MycN expression was observed in IMR-32 cells (Fig. 9).

### Comparable efficacy of TIST and RIST treatment despite a thousand-fold reduced mTOR inhibitor Torin-2 concentration

**Figure 10:**
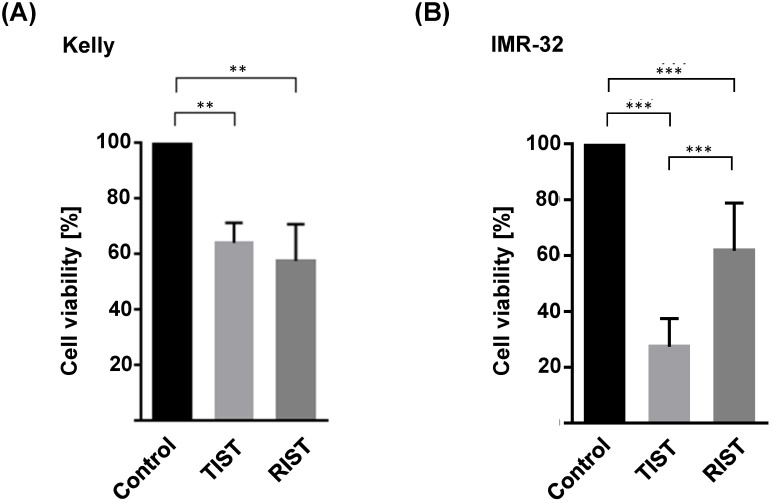
Viability test of multimodal drug treatment applying the RIST or TIST protocol in neuroblastoma cell line (A) Kelly and (B) IMR-32. Control: Vehicle (DMSO)-treated cells. Cells were seeded and treated with RIST, respectively TIST combination therapy as shown in Figure 10. Cells were seeded 24 h before drug treatment with a combination of Dasatinib with Torin-2 or Rapamycin, respectively. Drug concentrations used for the combination treatments in Kelly [D+T (TIST) = 1.5 μM + 9 nM; D+R (RIST) = 5 μM + 2 μM; SN-38+TMZ = 1 nM + 225 μM] and IMR-32 [D+T (TIST) = 1 μM + 6 nM; D+R = 0.05 μM + 0.13 μM; SN-38+TMZ = 0.4nM + 120 μM]. At day 8, the cell viability was tested by applying the MTT test as described under Methods. The cell viability was normalized to control-treated cells.

The multimodal RIST treatment protocol includes pre-treatment with the molecular targeted drugs Dasatinib and Rapamycin followed by treatment with the chemotherapeutics Irinotecan and Temozolomide (Fig. S4 and S5). To compare the RIST protocol with the alternative TIST protocol - replacing the allosteric mTOR inhibitor Rapamycin with the ATP competitive mTOR inhibitor Torin-2 - we first carried out an MTT assay to measure the cell viability. The *in vitro* treatment protocol is represented graphically (Fig. 12). All drugs were applied in IC_50_ concentrations of the corresponding combination treatments as listed in Table 4. Both multimodal drug treatment protocols showed a comparable inhibition of cell viability with 57.6 % for RIST and 64.1 % for TIST treatment in Kelly (Fig. 10) compared to control treated cells. In IMR-32, the inhibition of cell viability by TIST was significantly stronger (27.3%) compared to RIST treatment (61.7%) (Fig. 10).

**Figure 11:**
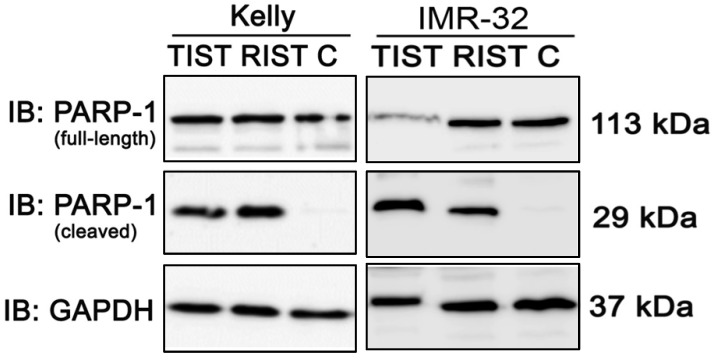
Immunoblot (IB) analysis of apoptotic marker expression (PARP cleavage) after multimodal drug treatment applying the RIST or TIST protocol, respectively, in neuroblastoma cell line Kelly and IMR-32. C: Vehicle (DMSO)-treated control cells. GAPDH served as loading control. Drug concentrations used for the combination treatment in Kelly [D+T (TIST) = 1.5 μM + 9 nM; D+R (RIST) = 5 μM + 2 μM; SN-38+TMZ = 1 nM + 225 μM] and IMR-32 [D+T (TIST) = 1 μM + 6 nM; D+R = 0.05 μM + 0.13 μM; SN-38+TMZ = 0.4 nM + 120 μM]. At day 8 cells were harvested and cell lysates were analyzed.

**Figure 12:**
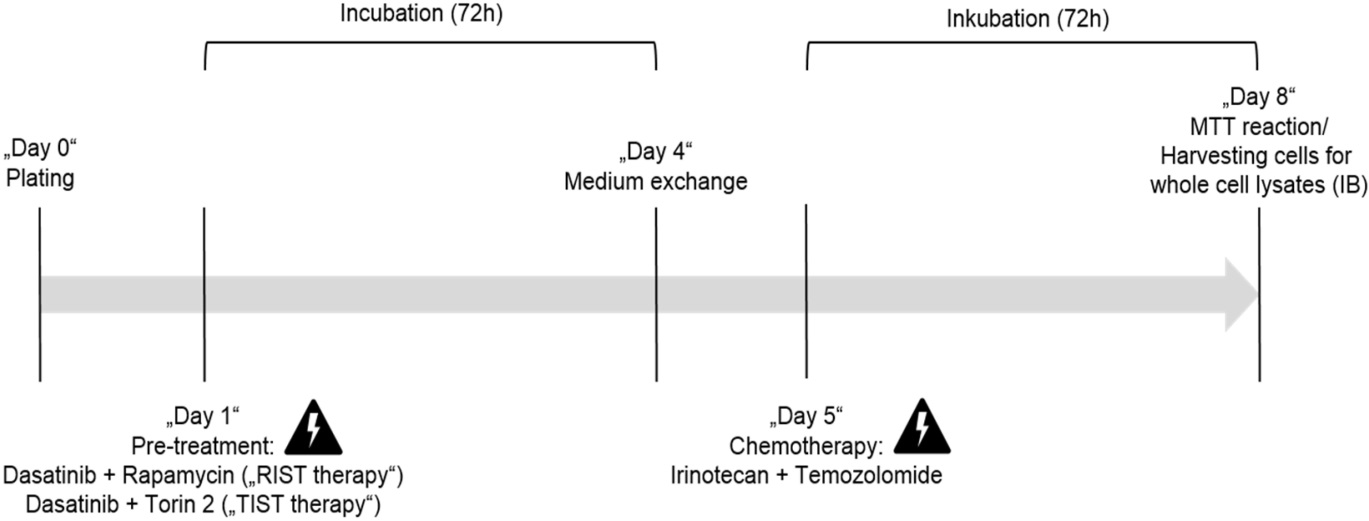
*In vitro* protocol for the multimodal RIST, respectively TIST treatment. Pre-treatment with the drug combination Dasatinib + Rapamycin for RIST respectively Dasatinib + Torin-2 for TIST for 72 h. Thereafter the medium was changed and 24h later the drug combination of conventional chemotherapeutics Irinotecan (SN-38) + Temozolomide (TMZ) was applied for additional 72 h.

We also investigated the induction of apoptosis by PARP-1 cleavage after both multimodal treatment protocols using immunoblot (Fig. 11). Compared to the control, both treatment protocols showed a marked cleavage of PARP-1 in NB cell line Kelly and IMR-32, indicating effective induction of apoptosis by both RIST and TIST treatment in neuroblastoma cells. In IMR-32 the induction of apoptosis by TIST treatment was even stronger compared to RIST as shown by increased PARP-1 cleavage products and - as a result of the strong cleavage - a marked decrease in full-length protein.

Taken together, although the reduction in cell viability as well as induction of apoptosis was comparable upon RIST or TIST treatment, it is important to note that the treatment concentration of Torin-2 is a thousand-fold lower compared to Rapamycin.

## Discussion

Targeting the PI3K/AKT/mTOR signaling pathway exhibits promising efficacy against human cancer and has been tested in various clinical trials, however, the complexity of the signaling network involving feedback loops and compensatory pathways, and intrinsic and acquired resistance limits the therapeutic success but may be addressed with combination treatments 43,44. Specifically for neuroblastoma numerous studies have shown that targeting the PI3K/AKT/mTOR signaling pathway is a valid treatment option for aggressive neuroblastoma and decreased proliferation *in vitro* and reduced tumor growth *in vivo* 17-19,41,42,45-48.

The limited success of Rapamycin as an anti-cancer drug led to the development of ATP competitive mTOR inhibitors such as Torin-2 30,31. Herein we demonstrate that the treatment of neuroblastoma cells with Torin-2 alone or in combination with Dasatinib have several advantages over Rapamycin treatment. Whether our results suggest that the replacement of Rapamycin by Torin-2 may improve the RIST treatment protocol will be discussed.

First, the IC_50_ of Torin-2 is reduced by more than a thousand-fold compared to Rapamycin (Table 1) which may result in a decrease of the required dose in clinical applications. Torin-2 is a highly selective mTOR inhibitor 49 with a 800-fold selectivity over PI3K 50 implicating that at low Torin-2 concentrations other kinases may only be minimally affected which also may result in reduced clinically relevant adverse events. Additionally, Torin-2 exhibits favorable pharmacokinetic properties. Compared to Torin-1, bioavailability, metabolic stability and plasma exposure was significantly improved 50. Moreover, it has been shown that Torin-2 induces a strong cytotoxic effect on T-ALL cells and stimulated T lymphocytes whereas it did not affect the viability of quiescent healthy CD4^+^ T lymphocytes 51. Therefore, Torin-2 rather preserves the immune system during molecular targeted anti-cancer therapy in contrast to the well-known immunosuppressive properties of Rapamycin that is broadly applied for prevention of acute rejection in transplant patients 36.

Aiming to illuminate the underlying cellular mechanism leading to impaired cell viability of neuroblastoma cells as observed in single as well as combination drug treatments, we found that cell cycle progression was inhibited. These results are well in line with Rapamycin and Torin-2 studies performed in other cancer cell types demonstrating reduced G_1_ to S cell cycle progression as well 36. We further analyzed potential mechanisms of the cell cycle arrest and detected a marked increase of p21 expression by both combination treatments (Fig. 6B). The overexpression of p21, an inhibitor of cyclin-dependent kinases, resulted in inhibition of proliferation in various types of cancer cells 52. Furthermore, p21 is associated with cellular senescence 53. It is assumed that senescence has an inhibitory effect on tumorigenesis and loss of senescence markers is associated with malignant progression 54. Therapy-induced senescence represents a new approach of anti-cancer treatment. It has been shown that senescent tumor cells are efficiently eliminated by immune cells migrating into the tumor leading to tumor regression 54.

Rapamycin is known to only incompletely inhibit mTORC1 while ATP competitive inhibitors were shown to additionally suppress these Rapamycin-resistant functions 23,24,55. In this study, combination treatment with Dasatinib/Rapamycin only slightly reduced phosphorylation of mTORC1 substrate 4E-BP while Dasatinib/Torin-2 potently suppressed 4E-BP phosphorylation (Fig. 8). By regulating the translation of specific mRNAs, 4E-BP strongly affects proliferation 56. The increased inhibition of mTORC1 by Torin-2 resulting in reduced phosphorylation of 4E-BP is likely to significantly contribute to the strong anti-proliferative effect of ATP competitive mTOR inhibitors 24,55.

Our study confirmed the known cell line specific mechanism of mTORC2 inhibition by Rapamycin after prolonged treatment (Fig. 8) 26. Interestingly, Rapamycin treatment induced even a slight hyper-phosphorylation of the mTORC2 substrate AKT (Ser473) at 6h post treatment compared to the control-treated cells whereas Torin-2 efficiently blocked the phosphorylation at this site (Fig. 8). This increased AKT phosphorylation may be caused by activation of PI3K signaling as a result of reduced negative feedback inhibition by Rapamycin-mediated inhibition of mTORC1 28,29. This AKT activation is likely to weaken the anti-tumor properties of Rapamycin by stimulating cancer promoting pathways 28. Furthermore, our data demonstrate that Torin-2 suppresses in addition the phosphorylation of AKT at Thr308 in cell line Kelly (Fig. 8). This phosphorylation site is a direct target of PDK-1 downstream of PI3K signaling 31. Phosphorylation of AKT at both sites is required for full activation of AKT 31,57,58. Studies have shown earlier that the inhibition of the phosphorylation of AKT at Ser473 by mTORC2 suppression also affects phosphorylation at Thr308 24,49,58,59. Feldman et al. 24 have shown that the reduced phosphorylation at Thr308 by an ATP competitive mTOR inhibitor (PP242) was dependent on the inhibition of Ser473 phosphorylation. A possible explanation for this might be that the inhibition of Ser473 phosphorylation transiently reduces the recruitment of AKT to the plasma membrane where AKT phosphorylation at Thr308 is catalyzed by the kinase PDK-1 49. Therefore, we conclude that reduced AKT Thr308 phosphorylation is a consequence of reduced Ser473 phosphorylation caused by Torin-2 induced mTORC2 inhibition as observed in both neuroblastoma cell lines (Fig. 8). In summary, the effective inhibition of mTORC2 by Torin-2 causes a strong suppression of cell survival, proliferation and metabolism promoting kinase AKT 28,31,60. Hence, replacing Rapamycin with Torin-2 may improve the anti-tumor property of the RIST treatment protocol.

Amplification of the oncogene MycN is frequent in neuroblastoma and is associated with a poor prognosis and rapid tumor progression 4,5. Hence, a therapy-induced decrease of MycN expression represents a promising approach for neuroblastoma therapy 40. Several studies have shown a decrease of MycN expression mediated by mTOR inhibitors 17,48. Our data demonstrate a reduction of MycN expression after both combination treatments in IMR-32 cells, whereas the MycN expression was not affected by any combination in Kelly cells (Fig. 9). This observed cell line-dependent impact of mTOR inhibition on MycN expression might be based on differences in expression of protein kinases like Aurora-A kinase or Anaplastic Lymphoma Kinase (ALK). Aurora A is known to prevent MycN degradation by protein:protein interaction 61. Thereby, neuroblastoma cells with high Aurora A expression are less dependent on growth factor and active PI3K/AKT signaling to maintain high MycN levels 61,62. Consequently, in Kelly cells the oncogene MycN may escape mTOR inhibition-mediated degradation by potentially high Aurora A expression. For a deeper understanding MycN expression should be examined in combination with an Aurora-A inhibitor that changes the conformation and prevents protein:protein interaction with MycN [for Aurora-A inhibitors: see 63]. On the other hand ALK is known to enhance the protein stability by reducing the phosphorylation of MycN at Thr58 62. In contrast to IMR-32, ALK is mutated in cell line Kelly (ALK^F1174L^) 64. Overexpression of ALK^F1174L^ has been shown to increase the oncogenic potential of MycN and led to early onset and lethality of disease 65. The protein stability of MycN was enhanced by ALK^F1174L^-mediated constitutive signaling 65. While Torin-2 induced ablation of MycN amplified tumors and reduction of MycN protein level, the ATP competitive mTOR inhibitor alone did not affect growth of tumors with both MycN amplification and ALK^F1174L^ mutation. Further, in those double mutated tumors, combination treatment with the ALK inhibitor Crizotinib and Torin-2 reduced tumor growth and partly initiated tumor regression 65. Therefore, in our study the ALK^F1174L^ mutation in Kelly cells is a likely cause of the failure to reduce MycN expression by the tested treatments with mTOR inhibitors Torin-2 and Rapamycin. In the future this assumption should be clarified by analyzing the MycN expression in Kelly cells treated with a combination of mTOR and ALK inhibitor such as Crizotinib.

Concluding, the combination treatment with Dasatinib and Torin-2 instead of Rapamycin revealed an increased mTOR pathway inhibition in neuroblastoma cells. In agreement, the alternative TIST treatment protocol inhibits cell viability and induces apoptosis despite a considerably reduced drug concentration. Our results suggest replacing Rapamycin by Torin-2 for the treatment protocol of relapsed or refractory high-risk neuroblastoma. However, *in vivo* testing in murine neuroblastoma models is critical to proof the validity of the TIST protocol and test for adverse effects.

## Supplementary Material

Supplementary figures and tables.

## Figures and Tables

**Table 1:** Viability test to determine half maximal inhibitory concentration (IC_50_) of various mTOR inhibitors in neuroblastoma (NB) cell line Kelly and IMR-32

NB cell line/mTOR inhibitor	Kelly [IC_50_]	IMR-32 [IC_50_]
**Rapamycin**	**27.21 ± 0.006 μM**	**37.47 ± 0.003 μM**
Torin-1	42.41 ± 0.017 nM	397.10 ± 0.037 nM
**Torin-2**	**11.69 ± 0.019 nM**	**29.67 ± 0.013 nM**
AZD3147	0.88 ± 0.015 nM	662.40 ± 0.033 nM
PP242	191.50 ±0.019 nM	390.50 ± 0.015 nM

**Note:** IC_50_ of Rapamycin = [24.27 ± 0.003 **μ**M] and Torin-2 = [28.52 ± 0.012 nM] in NB cell line SK-N-BE(2).

**Table 2:** Combinatorial viability tests to determine the combinatorial index (CI) of Dasatinib (D) combined with mTOR inhibitor Rapamycin (R) or Torin-2 (T) in neuroblastoma (NB) cell line Kelly, IMR-32, and SK-N-BE(2)

NB cell line/mTOR inhibitors	Kelly [CI]	IMR-32 [CI]	SK-N-BE(2) [CI]
Dasatinib/Rapamycin (D+R)	0.23 ± 0.07	0.20 ± 0.12	0.75 ± 0.02
Dasatinib/Torin-2 (D+T)	0.60 ± 0.15	0.17 ± 0.11	1.25 ± 0.11

**Note:** CI < 0.9 = synergistic; 0.9-1.1 = additive; > 1.1 = antagonistic

**Table 3:** Drug concentrations for single treatment

Drug/NB cell line	Kelly	IMR-32	SK-N-BE(2)
Rapamycin	27.2 µM	37.5 µM	24.3 µM
Torin-2	11.7 nM	29.7 nM	28.5 nM
Dasatinib	9.5 µM	1.5 µM	25.7 µM
Irinotecan (SN-38)	2.7 nM	0.7 nM	-
Temozolomide (TMZ)	246.0 µM	159.9 µM	-
Torin-1	42.4 nM	397.1 nM	-
AZD3147	0.9 nM	662.4 nM	-
PP242	191.5 nM	390.5 nM	-

**Table 4:** Drug concentrations for combination treatment

NB cell line/drug	Drug	Kelly	IMR-32	SK-N-BE(2)
Dasatinib/Rapamycin	Dasatinib [µM]	5.00	0.05	20.00
	Rapamycin [µM]	2.00	0.13	5.00
Dasatinib/Torin-2	Dasatinib [µM]	1.50	1.00	12.50
	Torin-2 [nM]	9.00	6.00	15.00
Irinotecan (SN-38)/ Temozolomide (TMZ)	Irinotecan [nM]	1.00	0.40	-
	Temozolomide [µM]	225.00	120.00	-

## References

[1] Maris JM, Hogarty MD, Bagatell R, Cohn SL (2007). Neuroblastoma. The Lancet.

[2] Kholodenko IV, Kalinovsky DV, Doronin II, Deyev SM, Kholodenko RV (2018). Neuroblastoma Origin and Therapeutic Targets for Immunotherapy. J Immunol Res.

[3] Brodeur GM (2003). Neuroblastoma: biological insights into a clinical enigma. Nat Rev Cancer.

[4] Brodeur GM, Seeger RC, Schwab M, Varmus HE, Bishop JM (1984). Amplification of N-myc in untreated human neuroblastomas correlates with advanced disease stage. Science.

[5] Schwab M, Westermann F, Hero B, Berthold F (2003). Neuroblastoma: biology and molecular and chromosomal pathology. The Lancet Oncology.

[6] Swift CC, Eklund MJ, Kraveka JM, Alazraki AL (2018). Updates in Diagnosis, Management, and Treatment of Neuroblastoma. Radiographics.

[7] Zage PE (2018). Novel Therapies for Relapsed and Refractory Neuroblastoma. Children (Basel).

[8] ClinicalTrials.gov. Multimodal Molecular Targeted Therapy to Treat Relapsed or Refractory High-risk Neuroblastoma - Full Text View - ClinicalTrials.gov: RIST-rNB-2011-Studienprotokoll

[9] Nonnenmacher L, Westhoff M-A, Fulda S, Karpel-Massler G, Halatsch M-E, Engelke J, Simmet T, Corbacioglu S, Debatin K-M (2015). RIST: A potent new combination therapy for glioblastoma. International Journal of Cancer.

[10] La Rosée P, Martiat P, Leitner A, Klag T, Müller MC, Erben P, Schenk T, Saussele S, Hochhaus A (2013). Improved tolerability by a modified intermittent treatment schedule of dasatinib for patients with chronic myeloid leukemia resistant or intolerant to imatinib. Ann Hematol.

[11] Bayat Mokhtari R, Homayouni TS, Baluch N, Morgatskaya E, Kumar S, Das B, Yeger H (2017). Combination therapy in combating cancer. Oncotarget.

[12] Hennessy BT, Smith DL, Ram PT, Lu Y, Mills GB (2005). Exploiting the PI3K/AKT pathway for cancer drug discovery. Nat Rev Drug Discov.

[13] Vivanco I, Sawyers CL (2002). The phosphatidylinositol 3-Kinase AKT pathway in human cancer. Nat Rev Cancer.

[14] Engelman JA (2009). Targeting PI3K signalling in cancer: opportunities, challenges and limitations. Nat Rev Cancer.

[15] Iżycka-Świeszewska E, Drożyńska E, Rzepko R, Kobierska-Gulida G, Grajkowska W, Perek D, Balcerska A (2010). Analysis of PI3K/AKT/mTOR signalling pathway in high risk neuroblastic tumours. Pol J Pathol.

[16] Opel D, Poremba C, Simon T, Debatin K-M, Fulda S (2007). Activation of Akt predicts poor outcome in neuroblastoma. Cancer Res.

[17] Johnsen JI, Segerström L, Orrego A, Elfman L, Henriksson M, Kågedal B, Eksborg S, Sveinbjörnsson B, Kogner P (2008). Inhibitors of mammalian target of rapamycin downregulate MYCN protein expression and inhibit neuroblastoma growth *in vitro* and *in vivo*. Oncogene.

[18] Segerström L, Baryawno N, Sveinbjörnsson B, Wickström M, Elfman L, Kogner P, Johnsen JI (2011). Effects of small molecule inhibitors of PI3K/Akt/mTOR signaling on neuroblastoma growth *in vitro* and *in vivo*. International Journal of Cancer.

[19] Chesler L, Schlieve C, Goldenberg DD, Kenney A, Kim G, McMillan A, Matthay KK, Rowitch D, Weiss WA (2006). Inhibition of phosphatidylinositol 3-kinase destabilizes Mycn protein and blocks malignant progression in neuroblastoma. Cancer Res.

[20] Rad E, Murray JT, Tee AR (2018). Oncogenic Signalling through Mechanistic Target of Rapamycin (mTOR): A Driver of Metabolic Transformation and Cancer Progression. Cancers (Basel).

[21] Loewith R, Jacinto E, Wullschleger S, Lorberg A, Crespo JL, Bonenfant D, Oppliger W, Jenoe P, Hall MN (2002). Two TOR Complexes, Only One of which Is Rapamycin Sensitive, Have Distinct Roles in Cell Growth Control. Molecular Cell.

[22] Huang K, Fingar DC (2014). Growing knowledge of the mTOR signaling network. Semin Cell Dev Biol.

[23] Thoreen CC, Sabatini DM (2009). Rapamycin inhibits mTORC1, but not completely. Autophagy.

[24] Feldman ME, Apsel B, Uotila A, Loewith R, Knight ZA, Ruggero D, Shokat KM (2009). Active-site inhibitors of mTOR target rapamycin-resistant outputs of mTORC1 and mTORC2. PLoS Biol.

[25] Choo AY, Yoon S-O, Kim SG, Roux PP, Blenis J (2008). Rapamycin differentially inhibits S6Ks and 4E-BP1 to mediate cell-type-specific repression of mRNA translation. Proc Natl Acad Sci U S A.

[26] Sarbassov DD, Ali SM, Sengupta S, Sheen J-H, Hsu PP, Bagley AF, Markhard AL, Sabatini DM (2006). Prolonged Rapamycin Treatment Inhibits mTORC2 Assembly and Akt/PKB. Molecular Cell.

[27] Sabatini DM (2006). mTOR and cancer: insights into a complex relationship. Nat Rev Cancer.

[28] O'Reilly KE, Rojo F, She Q-B, Solit D, Mills GB, Smith D, Lane H, Hofmann F, Hicklin DJ, Ludwig DL (2006). mTOR inhibition induces upstream receptor tyrosine kinase signaling and activates Akt. Cancer Res.

[29] Wan X, Harkavy B, Shen N, Grohar P, Helman LJ (2007). Rapamycin induces feedback activation of Akt signaling through an IGF-1R-dependent mechanism. Oncogene.

[30] Xie J, Wang X, Proud CG (2016). mTOR inhibitors in cancer therapy. F1000Res.

[31] Zoncu R, Efeyan A, Sabatini DM (2011). mTOR: from growth signal integration to cancer, diabetes and ageing. Nat Rev Mol Cell Biol.

[32] King D, Yeomanson D, Bryant HE (2015). PI3King the lock: targeting the PI3K/Akt/mTOR pathway as a novel therapeutic strategy in neuroblastoma. Journal of Pediatric Hematology/Oncology.

[33] Morgan DML (1998). Tetrazolium (MTT) Assay for Cellular Viability and Activity. In: Morgan DML, editor. Polyamine Protocols. Totowa, NJ: Humana Press.

[34] Chou T-C, Talalay P (1984). Quantitative analysis of dose-effect relationships: the combined effects of multiple drugs or enzyme inhibitors. Advances in Enzyme Regulation.

[35] Bijnsdorp IV, Giovannetti E, Peters GJ (2011). Analysis of drug interactions. Methods Mol Biol.

[36] Sehgal SN (2003). Sirolimus: its discovery, biological properties, and mechanism of action. Transplant Proc.

[37] Kim YC, Guan K-L (2015). mTOR: a pharmacologic target for autophagy regulation. J Clin Invest.

[38] Mizushima N, Yoshimori T (2007). How to Interpret LC3 Immunoblotting. Autophagy.

[39] Mathiassen SG, Zio D de, Cecconi F (2017). Autophagy and the Cell Cycle: A Complex Landscape. Front Oncol.

[40] Barone G, Anderson J, Pearson ADJ, Petrie K, Chesler L (2013). New strategies in neuroblastoma: Therapeutic targeting of MYCN and ALK. Clin Cancer Res.

[41] Chanthery YH, Gustafson WC, Itsara M, Persson A, Hackett CS, Grimmer M, Charron E, Yakovenko S, Kim G, Matthay KK (2012). Paracrine signaling through MYCN enhances tumor-vascular interactions in neuroblastoma. Sci Transl Med.

[42] Cage TA, Chanthery Y, Chesler L, Grimmer M, Knight Z, Shokat K, Weiss WA, Gustafson WC (2015). Downregulation of MYCN through PI3K Inhibition in Mouse Models of Pediatric Neural Cancer. Front Oncol.

[43] Fruman DA, Chiu H, Hopkins BD, Bagrodia S, Cantley LC, Abraham RT (2017). The PI3K Pathway in Human Disease. Cell.

[44] Yang J, Nie J, Ma X, Wei Y, Peng Y, Wei X (2019). Targeting PI3K in cancer: mechanisms and advances in clinical trials. Mol Cancer.

[45] Erdreich-Epstein A, Singh AR, Joshi S, Vega FM, Guo P, Xu J, Groshen S, Ye W, Millard M, Campan M (2017). Association of high microvessel αvβ3 and low PTEN with poor outcome in stage 3 neuroblastoma: rationale for using first in class dual PI3K/BRD4 inhibitor, SF1126. Oncotarget.

[46] Mohlin S, Hamidian A, Stedingk K von, Bridges E, Wigerup C, Bexell D, Påhlman S (2015). PI3K-mTORC2 but not PI3K-mTORC1 regulates transcription of HIF2A/EPAS1 and vascularization in neuroblastoma. Cancer Res.

[47] Stewart E, Shelat A, Bradley C, Chen X, Federico S, Thiagarajan S, Shirinifard A, Bahrami A, Pappo A, Qu C (2015). Development and characterization of a human orthotopic neuroblastoma xenograft. Dev Biol.

[48] Vaughan L, Clarke PA, Barker K, Chanthery Y, Gustafson CW, Tucker E, Renshaw J, Raynaud F, Li X, Burke R (2016). Inhibition of mTOR-kinase destabilizes MYCN and is a potential therapy for MYCN-dependent tumors. Oncotarget.

[49] Liu Q, Xu C, Kirubakaran S, Zhang X, Hur W, Liu Y, Kwiatkowski NP, Wang J, Westover KD, Gao P (2013). Characterization of Torin2, an ATP-competitive inhibitor of mTOR, ATM, and ATR. Cancer Res.

[50] Liu Q, Wang J, Kang SA, Thoreen CC, Hur W, Ahmed T, Sabatini DM, Gray NS (2011). Discovery of 9-(6-aminopyridin-3-yl)-1-(3-(trifluoromethyl)phenyl)benzoh1,6naphthyridin-2(1H)-one (Torin2) as a potent, selective, and orally available mammalian target of rapamycin (mTOR) inhibitor for treatment of cancer. J Med Chem.

[51] Alameen AAM, Simioni C, Martelli AM, Zauli G, Ultimo S, McCubrey JA, Gonelli A, Marisi G, Ulivi P, Capitani S (2016). Healthy CD4+ T lymphocytes are not affected by targeted therapies against the PI3K/Akt/mTOR pathway in T-cell acute lymphoblastic leukemia. Oncotarget.

[52] Gartel AL, Tyner AL (1998). The growth-regulatory role of p21 (WAF1/CIP1). Prog Mol Subcell Biol.

[53] Chang BD, Xuan Y, Broude EV, Zhu H, Schott B, Fang J, Roninson IB (1999). Role of p53 and p21waf1/cip1 in senescence-like terminal proliferation arrest induced in human tumor cells by chemotherapeutic drugs. Oncogene.

[54] Collado M, Serrano M (2010). Senescence in tumours: evidence from mice and humans. Nat Rev Cancer.

[55] Thoreen CC, Kang SA, Chang JW, Liu Q, Zhang J, Gao Y, Reichling LJ, Sim T, Sabatini DM, Gray NS (2009). An ATP-competitive mammalian target of rapamycin inhibitor reveals rapamycin-resistant functions of mTORC1. J Biol Chem.

[56] Dowling RJO, Topisirovic I, Alain T, Bidinosti M, Fonseca BD, Petroulakis E, Wang X, Larsson O, Selvaraj A, Liu Y (2010). mTORC1-mediated cell proliferation, but not cell growth, controlled by the 4E-BPs. Science.

[57] Alessi DR, Andjelkovic M, Caudwell B, Cron P, Morrice N, Cohen P, Hemmings BA (1996). Mechanism of activation of protein kinase B by insulin and IGF-1. The EMBO Journal.

[58] Guertin DA, Sabatini DM (2007). Defining the Role of mTOR in Cancer. Cancer Cell.

[59] Rodrik-Outmezguine VS, Chandarlapaty S, Pagano NC, Poulikakos PI, Scaltriti M, Moskatel E, Baselga J, Guichard S, Rosen N (2011). mTOR kinase inhibition causes feedback-dependent biphasic regulation of AKT signaling. Cancer Discov.

[60] Sparks CA, Guertin DA (2010). Targeting mTOR: prospects for mTOR complex 2 inhibitors in cancer therapy. Oncogene.

[61] Otto T, Horn S, Brockmann M, Eilers U, Schüttrumpf L, Popov N, Kenney AM, Schulte JH, Beijersbergen R, Christiansen H (2009). Stabilization of N-Myc is a critical function of Aurora A in human neuroblastoma. Cancer Cell.

[62] Otto T (2015). MYCN and Its Posttranslational Regulation in Neuroblastoma. In: Christiansen H, Christiansen NM, editors. Progressive Neuroblastoma.

[63] Gustafson WC, Meyerowitz JG, Nekritz EA, Chen J, Benes C, Charron E, Simonds EF, Seeger R, Matthay KK, Hertz NT (2014). Drugging MYCN through an allosteric transition in Aurora kinase A. Cancer Cell.

[64] Harenza JL, Diamond MA, Adams RN, Song MM, Davidson HL, Hart LS, Dent MH, Fortina P, Reynolds CP, Maris JM (2017). Transcriptomic profiling of 39 commonly-used neuroblastoma cell lines. Sci Data.

[65] Berry T, Luther W, Bhatnagar N, Jamin Y, Poon E, Sanda T, Pei D, Sharma B, Vetharoy WR, Hallsworth A (2012). The ALK(F1174L) mutation potentiates the oncogenic activity of MYCN in neuroblastoma. Cancer Cell.

