# Supplementary material for: Comparing mTOR inhibitor Rapamycin with Torin-2 within the RIST molecular-targeted regimen in neuroblastoma cells
Source: Int J Med Sci. 2021 Jan 1;18(1):137–49. doi: 10.7150/ijms.48393 (PMC7738968; doi:10.7150/ijms.48393)
Supplement: Supplementary file 1 — Supplementary figures and tables. [file ijmsv18p0137s1.pdf]

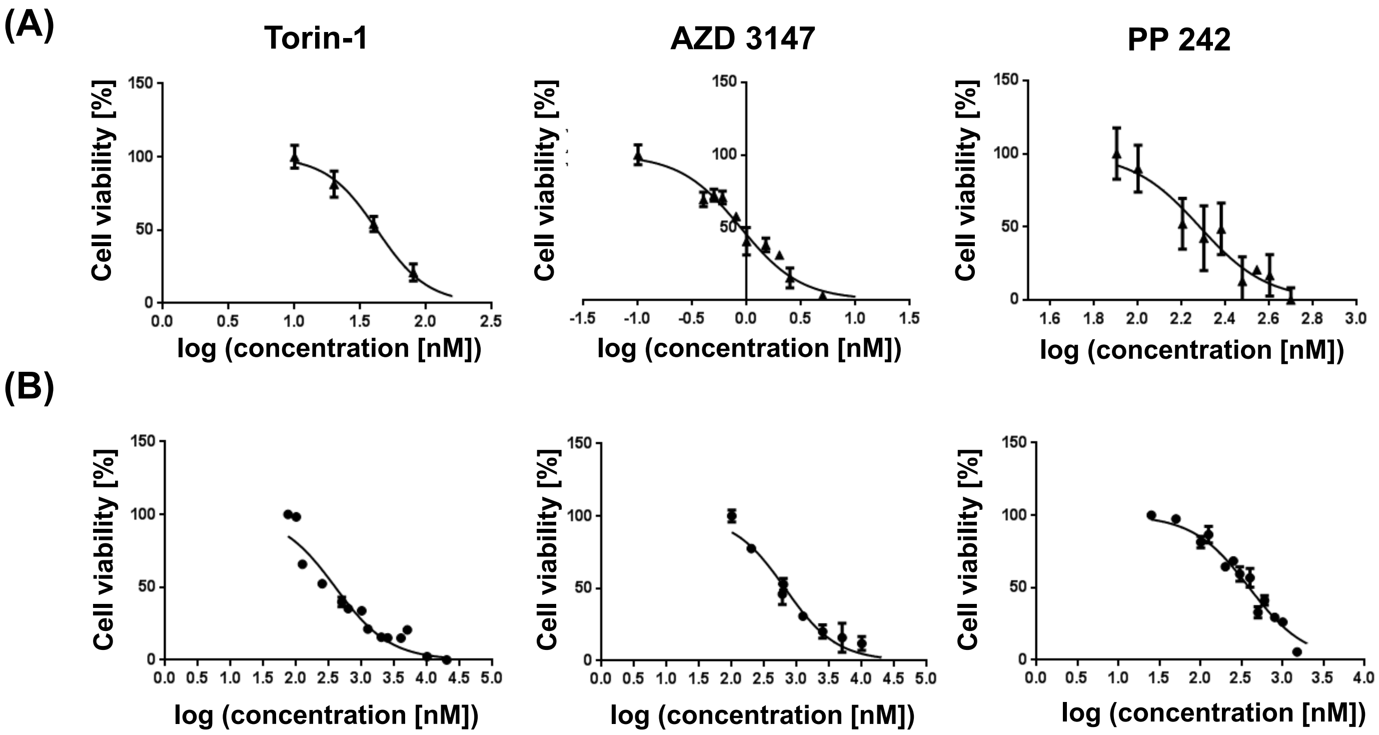

Fig. S1: Viability tests to determine the IC_{50} value of various mTOR inhibitors in neuroblastoma cell line (A) Kelly and (B) IMR-32 after 72h treatment. The cell viability was tested by applying the MTT test as described under Methods.

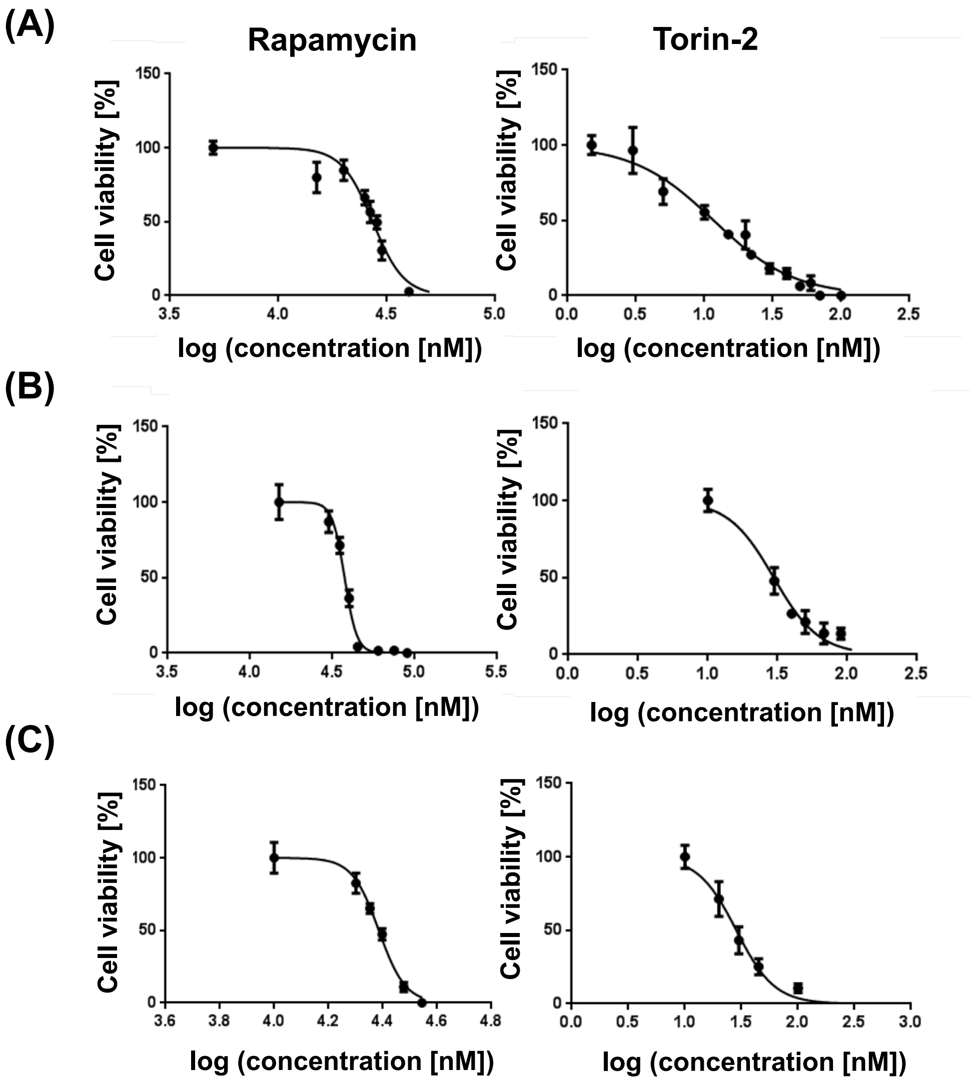

Fig. S2: Viability tests to determine IC_{50} of Rapamycin and Torin-2 in neuroblastoma cell line (A) Kelly, (B) IMR-32, and (C) SK-N-BE(2) after 72h treatment. The cell viability was tested by applying the MTT test as described under Methods.

(A)

Dasatinib

Cell viability [%]

log (concentration [nM])

(B)

Cell viability [%]

log (concentration [nM])

(C)

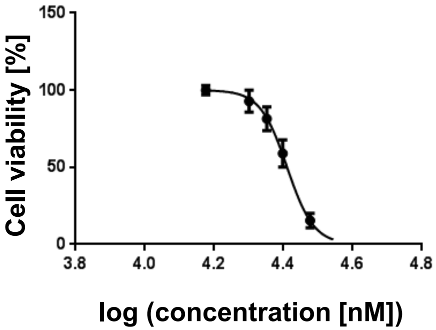

Fig. S3: Viability tests to determine IC_{50} of Dasatinib in neuroblastoma cell line (A) Kelly, (B) IMR-32, and (C) SK-N-BE(2) after 72h treatment. The cell viability was tested by applying the MTT test as described under Methods.

Table S1: Viability tests to determine IC_{50} of Dasatinib in neuroblastoma (NB) cell line Kelly, IMR-32, and SK-N-BE(2).

**NB cell line**	**Dasatinib** [μM]
**Kelly**	9.47 ± 0.010
**IMR-32**	1.53 ± 0.053
**SK-N-BE(2)**	25.73 ± 0.003

Table S2: Viability tests to determine the dose reduction factor (DRF) of Dasatinib combined with Rapamycin in neuroblastoma (NB) cell line Kelly, IMR-32, and SK-N-BE(2).

mTOR-inhibitor/ NB cell line	Dasatinib [μM]	Rapamycin [μM]	Dasatinib [DRF]	Rapamycin [DRF]
Kelly	5.00	2.00	1.9	13.6
IMR-32	0.05	0.13	30.6	288.2
SK-N-BE(2)	20.00	5.00	1.3	4.9

Table S3: Viability tests to determine the dose reduction factor (DRF) of Dasatinib combined with Torin-2 in neuroblastoma (NB) cell line Kelly, IMR-32, and SK-N-BE(2).

mTOR-inhibitor/ NB cell line	Dasatinib [μM]	Torin-2 [nM]	Dasatinib [DRF]	Torin-2 [DRF]
Kelly	1.50	9.00	6.3	1.3
IMR-32	1.00	6.00	1.5	4.9
SK-N-BE(2)	12.50	15.00	2.1	1.9

Table S4: Viability test to determine half maximal inhibitory concentration (IC_{50}) of molecular-targeted inhibitors Rapamycin, Torin-2, and Dasatinib as well as chemotherapeutics Irinotecan (SN-38) and Temozolomide (TMZ) in neuroblastoma (NB) cell line Kelly and IMR-32.

Inhibitor/ NB cell line	Rapamycin [µM]	Dasatinib [µM]	Torin-2 [nM]	SN-38 [nM]	TMZ [µM]
**Kelly**	27.21 ± 0.006	9.47 ± 0.010	11.69 ± 0.019	2.74 ± 0.008	246.00 ± 0.004
**IMR-32**	37.47 ± 0.003	1.53 ± 0.057	29.67 ± 0.013	0.67 ± 0.009	159.90 ± 0.004

Table S5: Viability tests to determine the dose reduction factor (DRF) of Irinotecan (SN-38) combined with Temozolomide (TMZ) in neuroblastoma (NB) cell line Kelly and IMR-32.

mTor-inhibitor/ NB cell line	SN-38 [nM]	TMZ [µM]	SN-38 [DRF]	TMZ [DRF]
**Kelly**	1.00	225.00	2.7	1.1
**IMR-32**	0.40	120.00	0.2	1.3

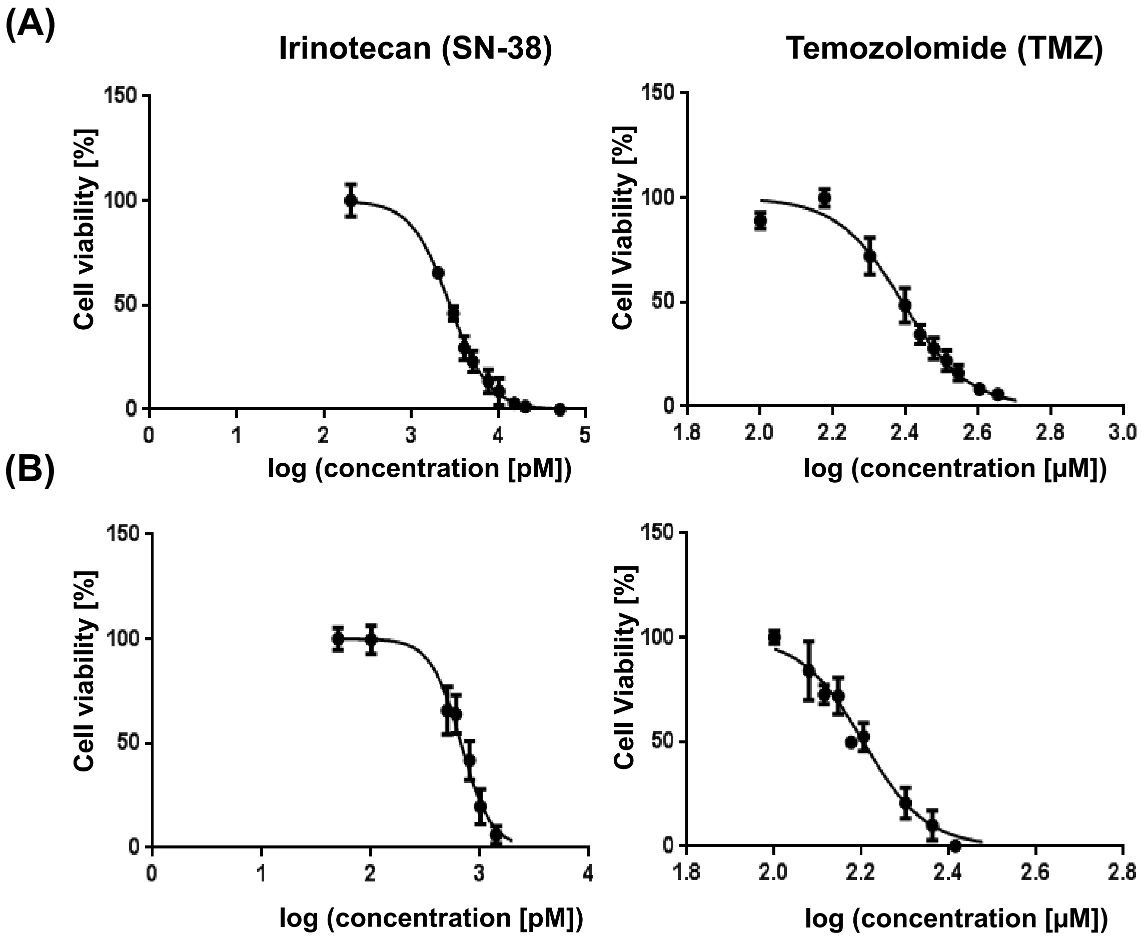

Fig. S4: Viability tests to determine IC_{50} of Irinotecan (SN-38) and Temozolomide (TMZ) in neuroblastoma cell line (A) Kelly and (B) IMR-32 after 72h treatment. The cell viability was tested by applying the MTT test as described under Methods.

(A) Kelly (B) IMR-32

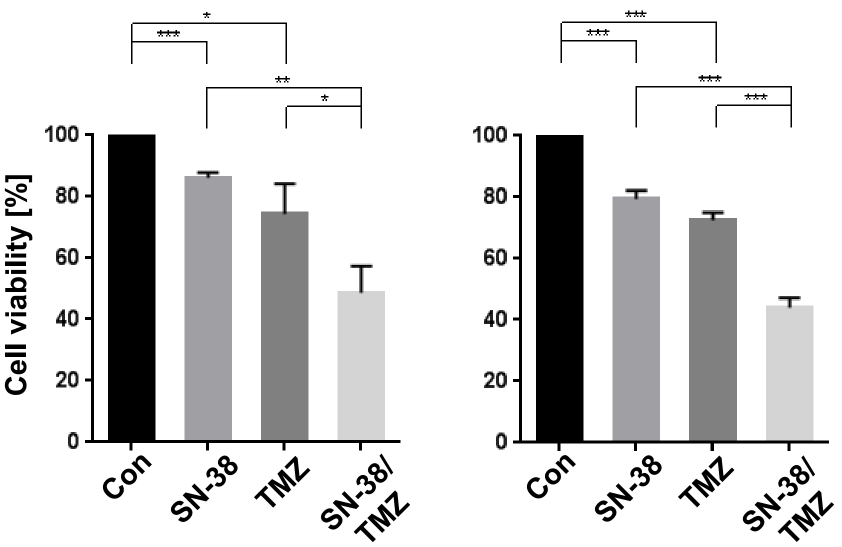

(C)

Inhibitor/<br>NB cell line	SN-38 + TMZ<br>[CI]
**Kelly**	1.20 ± 0.09
**IMR-32**	1.30 ± 0.15

Note: CI < 0.9 = synergistic; 0.9-1.1 = additive; > 1.1 = antagonistic

Fig. S5: Viability tests to determine IC_{50} of chemotherapeutics Irinotecan (SN-38), Temozolomide (TMZ), and drug combination (SN-38/TMZ) in neuroblastoma (NB) cell line (A) Kelly and (B) IMR-32. Con: DMSO-treated control cells. (C) Viability tests to determine the combinatorial index (CI). The cell viability was tested by applying the MTT test as described under Methods.
